# Longitudinal Study on Liver Functions in Patients with Thalassemia Major before and after Deferasirox (DFX) Therapy

**DOI:** 10.4084/MJHID.2014.025

**Published:** 2014-04-07

**Authors:** Ashraf Soliman, Mohamed Yassin, Fawzia Al Yafei, Lolwa Al-Naimi, Noora Almarri, Aml Sabt, Vincenzo De Sanctis

**Affiliations:** 1 Department of Pediatrics, Alexandria University Children’s Hospital, Alexandria, Egypt; 2 Departments of Pediatrics, Hamad Medical Center (HMC), Doha – Qatar; 3 Departments of Hematology, Hamad Medical Center (HMC), Doha – Qatar; 4 Pediatric and Adolescent Outpatient Clinic, Quisisana Hospital, 44121 Ferrara, Italy.

## Abstract

By performing regular blood transfusion and iron chelation therapy, most patients with beta thalassemia major (BTM) now survive beyond the third decade of life. Liver disease is becoming an important cause of morbidity and mortality in these patients. Chronic hepatitis and/or severe iron overload are both important causes of liver pathology. Iron chelation with desferrioxamine (DFO) reduces excessive body iron, but its efficacy is limited by poor compliance and dose related toxicity. The recent use of Deferasirox ( DFX ), an oral single dose therapy, has improved the compliance to chelation.

**Aims:**

To study the long-term liver functions in BMT patients, seronegative for liver infections before versus after DFX treatment in relation to ferritin level.

**Methods:**

Only BTM patients with hepatitis negative screening (checked every year) and on treatment with DFO for at least five years and with DFX for four years were enrolled. Liver function tests including serum bilirubin, alanine transferase (ALT), aspartate transferase (AST), albumin, insulin-like growth factor – I (IGF-I) and serum ferritin concentrations were followed every six months in 40 patients with BTM.

**Results:**

DFX treatment (20 mg/kg/day) significantly decreased serum ferritin level in patients with BTM; this was associated with a significant decrease in serum ALT, AST, ALP and increase in IGF-I concentrations. Albumin concentrations did not change after DFX treatment. ALT and AST levels were correlated significantly with serum ferritin concentrations ( r = 0.45 and 0.33 respectively, p < 0.05). IGF-I concentrations were correlated significantly with serum ALT (r= 0.26, p = 0.05) but not with AST, ALP, bilirubin or albumin levels.

The negative correlation between serum ferritin concentrations and ALT suggests that the impairment of hepatic function negatively affect IGF-I synthesis in these patients due to iron toxicity, even in the absence of hepatitis.

**Conclusions:**

Some impairment of liver function can occur in hepatitis negative thalassemic patients with iron overload. The use of DFX was associated with mild but significant reduction of ALT, AST and ALP and increase in IGF-I levels. The negative correlation between IGF-I and ALT concentrations suggest that preventing hepatic dysfunction may improve the growth potential in these patients.

## Introduction

The β and α thalassaemias are the most common inherited single-gene disorders in the world. Iron overload is a consequence of chronic transfusion therapy that adversely affects the function of the heart, liver and endocrine glands. Even with the administration of effective subcutaneous (s.c.) iron chelation therapy with desferrioxamine (DFO), over 50% of patients die before the age of 35 years, mainly because of poor compliance with s.c. chelation regimens.^1^

A high prevalence of hepatic hemosiderosis (grades 3–4) has been recorded in many studies.^2^–^4^ Hepatic fibrosis is also still not uncommon in patients with β thalassemia major (BTM) despite the use of chelation therapy. This could reflect the rather unsatisfactory compliance rate with DFO treatment observed in many of BTM patients. Thus, early and accurate diagnosis of liver disease followed by prompt intervention may prevent liver disease progression.^2^–^4^

The liver is the primary site of iron storage and the only site for synthesis of transferrin and ferritin. Free ferrous iron is highly toxic and normally is protein-bound within the liver. Unbound, iron catalyzes the production of free radicals, which have been implicated in lipid peroxidation and hepatotoxicity. Lipid peroxidation may be the primary event causing hepatocellular injury secondary to iron overload.^5^–^8^

Significant correlation between ferritin iron concentration and individual liver iron concentration, measured non-invasively by superconducting quantum interference device biomagnetometry (SQUID) has been reported in patients with BTM and hemochromatosis. However, the relation between serum ferritin concentration and liver iron improves when serum ferritin is lower than 2500 μg/and in the absence of hepatitis.^5^–^8^ In a large cohort of patients on chronic transfusion, a strong statistical correlation has been found between liver histology, serum ferritin and liver iron content (LIC).^9^

In addition, patients with thalassemia have a high prevalence of hepatitis B and C infections.^10^–^12^ HCV infected patients had significantly higher enzymes than non-infected.^13^ chronic hepatitis C virus infection has been associated with liver iron loading. The cause of elevated serum iron indices in some HCV-infected individuals is not clear. The concomitant increase of in serum alanine aminotransferase (ALT) levels suggests that iron and ferritin be released from damaged hepatocytes as a result of hepatic necro-inflammation.^14^ In addition, increased iron has been shown to enhance HCV replication in vitro.^15^ Furthermore, hyperferritinemia and increased iron stores have been associated with the severity of liver damage in non-alcoholic fatty liver diseases (NAFLD), and iron depletion reduced insulin resistance and liver enzymes. Serum ferritin concentration is an important determinant of liver enzyme levels, and increased serum ferritin level is an independent predictor of liver damage in these patients, so it is useful to identify patients at risk of steatohepatitis and advanced fibrosis.^16^–^21^ Histological evidence of hepatic iron accumulation has also been associated with an increased risk of fibrosis in large multicenter studies, in patients with NAFLD both from Europe and the United States. The β globin mutations, the best predictor of parenchymal iron overload in the Mediterranean area, are associated with almost double risk of severe fibrosis.^20^–^24^

These data suggest that incorporation of serum ferritin level can improve the performance of noninvasive scoring of liver damage in patients with chronic liver disease and that iron depletion still represents an attractive therapeutic target to prevent the progression of liver damage in these patients.^25^ Experimental evidence suggests that iron depletion induced by chelators induce glucose uptake and utilization in hepatocytes in vitro and in vivo liver, increasing insulin receptor binding activity and signaling.^26^,^27^

Randomized and controlled trials have established that the oral deferasirox ( DFX) efficacy is comparable to the standard iron chelator, DFO administered as a parenteral infusion, in reducing liver iron concentration and serum ferritin levels. However, DFX may be more effective than DFO in actual clinical practice owing to the improvement in quality of life and, hence, increased compliance associated with the oral route of administration.^28^–^30^

We investigated and reviewed the liver function in 40 BMT patients attending the Hematology Clinic of Hamad Medical Center, Doha (Qatar) during follow-up of 10 years, in order to ascertain the relationship, between serum ferritin concentrations and different liver functions, before and after DFX therapy.

## Patients and Methods

The study was designed on the basis of observational study. Subjects were randomly recruited from the hematology and endocrinology clinics of HMC Doha (Qatar) and analyzed in the Biochemistry Laboratory of HMC. A detailed history including the age at diagnosis of BMT and clinical presentation and transfusion and chelation data was taken from the patient, mother or the attendant. The ethical committee of Hamad Medical Center has approved the study protocol as a part of protocol for studying the endocrine and biochemical functions in thalassemic patients. Waiver of informed consent was taken for accessing data of patients before inclusion in the study. The diagnosis of BMT was confirmed by Hb-electrophoresis in all the patients.

The patients who fulfilled the inclusion criteria were incorporated in the study. All the planned information were obtained and recorded in the data collecting sheet properly. A total of 45 subjects were included in the study. All but five patients did not complete the study (three left the city and two had splenectomy).

## Inclusion and Exclusion Criteria

Patients with a confirmed diagnosis of BTM above the age of 5 years were randomly selected. All the subjects were on regular blood transfusions and iron chelation using subcutaneous pump infusion of DFO five days per week. Exclusion criteria included: (1) Thalassemia trait or intermedia type, (2) History of jaundice due to viral hepatitis (3) History of splenectomy, (4) Positive screening test for hepatitis C or B.

Only patients with hepatitis negative screening (checked every year), and on treatment with DFO for at least five years or on treatment with DFX for four years or more were enrolled.

Liver function tests including serum bilirubin, ALT, aspartate transferase (AST), albumin, insulin-like growth factor – I (IGF-I) and serum ferritin concentrations were followed every six months in all these patients,

## Statistical Analysis

Variables including age, serum ferritin, bilirubin, ALT, AST, ALP, and IGF-I concentrations are expressed as mean +/− standard deviation. Comparison of variables before versus after DFX treatment was performed using Student’s t test or analysis of variance as appropriate.

The possible associations between serum ferritin and different liver functions are tested using linear regression equation. The level of significance was set at 0.05 in the analyses, and all the statistical testing was two sides.

## Results

Forty patients with BTM were evaluated longitudinally every six months from age of eight to 18 years. Their mean age at the beginning of the study was 6.8 +/− 1.2 years and at the end of the study was 18.4 +/− 1.7 years.

They started iron chelation therapy with DFO at the age of 3.8 +/− 0.9 years. They were shifted to oral DFX (20 mg/kg/day) at the age of 13.8 +/− 1.5 years.

**Figure 1:**
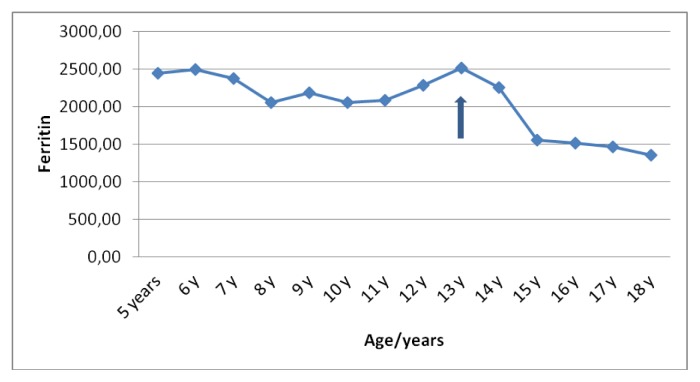
Serum ferritin concentrations (μg/L) before and after DFO therapy ( 
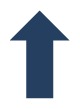
).

**Figure 2:**
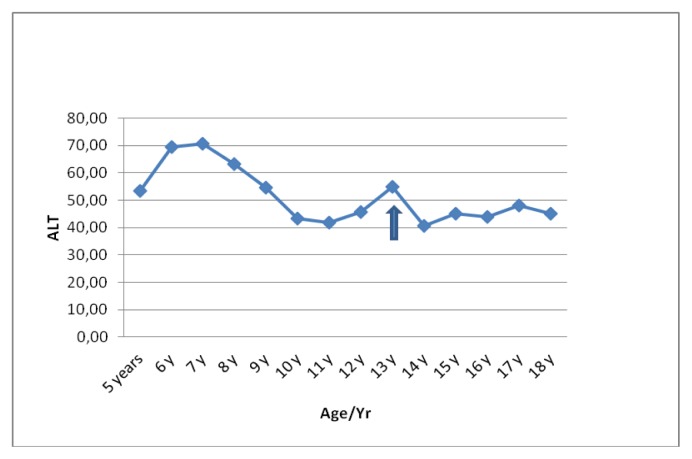
Serum ALT concentrations (U/L) before and after DFO therapy ( 
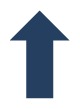
).

**Figure 3:**
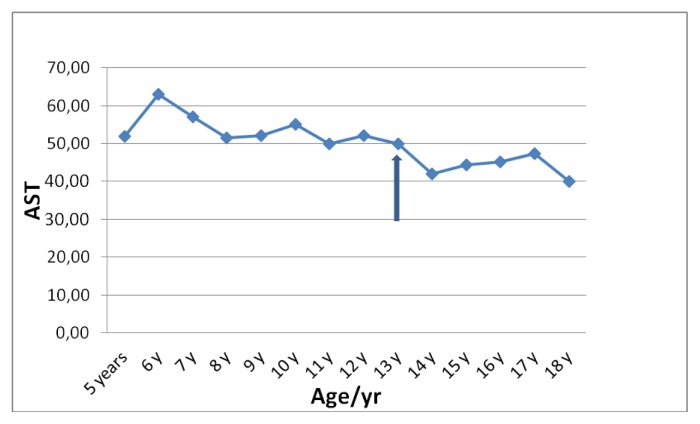
Serum AST concentrations **(**U/L) before and after DFO therapy ( 
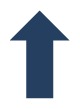
).

**Figure 4:**
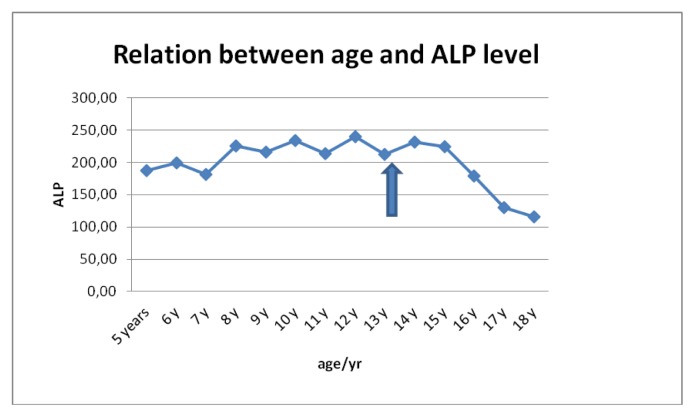
Serum ALP concentrations (U/L) before and after DFO therapy ( 
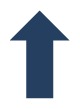
).

**Fig 5:**
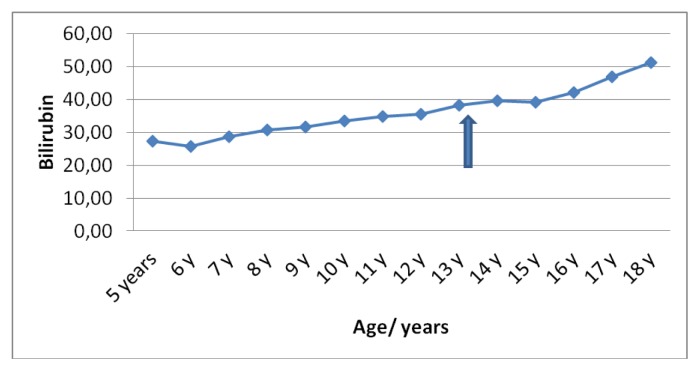
Serum bilirubin concentrations (μmol/L) before and after DFO therapy ( 
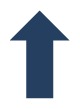
).

**Fig 6:**
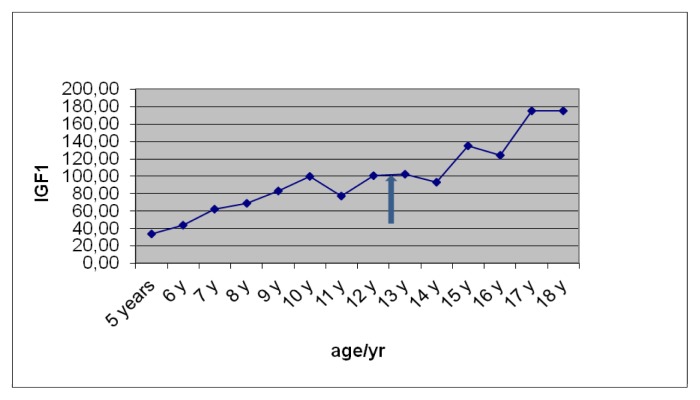
Serum IGF-I concentrations (ng/dl) before and after DFO therapy ( 
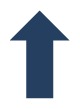
).

Liver functions followed longitudinally are presented in Table 1. After initiation of oral DFX, the serum ferritin level significantly decreased in all BTM patients (p < 0.001). This was associated with mild, but significant, decrease in serum ALT, AST and ALP concentrations and increase in IGF-I concentrations (p < 0.01). (Figure 1–6) Albumin concentrations did not change after treatment. There was a mild significant increase in serum bilirubin concentrations.

**Figure 7:**
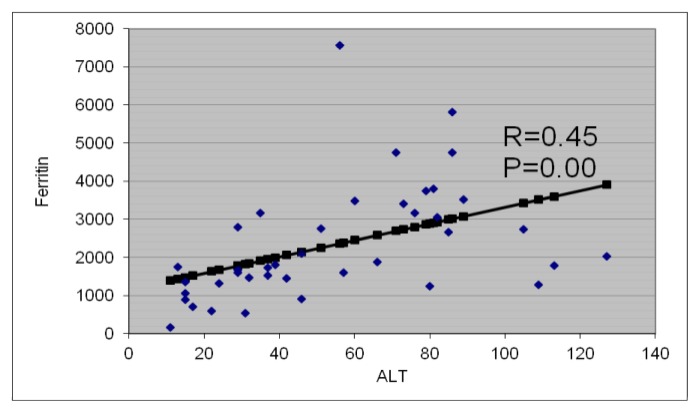
Correlation between ALT to serum ferritin concentrations in thalassemia patients.

ALT and AST levels were correlated significantly with serum ferritin concentrations (r = 0.45 and 0.33 respectively, p < 0.05) (Figure 7). IGF-I concentrations were correlated significantly with serum ALT (r = − 0.26, p = 0.05) and serum ferritin (r = −0.29, p = 0.02) concentrations. IGF-I concentrations were not correlated with AST, ALP, bilirubin or albumin levels (p > 0.05). (Table 2)

In our BMT patients with negative hepatitis screening, DFO treatment given at a younger age was not associated with significant hepatic dysfunction. However, the DFX treatment significantly induced a decrease of ALT, AST and ALP concentrations.

The negative correlation between serum ferritin concentrations and ALT suggests that the impairment of hepatic function negatively affect IGF-I synthesis in these patients due to iron overload, even in the absence of hepatitis.

## Discussion

In thalassemia, abnormal liver function appears to be related to the high ferritin levels and the age when transfusions was initiated.^5^–^9^

Iron-induced liver disease is often aggravated by viral infection. Hepatic siderosis, portal fibrosis and even cirrhosis may develop despite iron chelation therapy.^13^–^15^ Elevated serum ALT levels should alert the clinician about the possibility of hepatitis due to multiple blood transfusions.^31^,^32^

In this longitudinal study liver, function tests and serum ferritin levels were observed for more than 10 years in patients with BTM with negative screening for hepatitis C and B in order to exclude the possible effects of hepatitis in the production determination of liver dysfunction. They started iron chelation therapy with DFO at the age of 3.8 +/− 0.9 years then were shifted to oral DFX (20 mg/kg/day) at the age of 13.8 +/− 1.5 years.

Liver functions, followed longitudinally, showed that the DFX treatment decreased serum ferritin level significantly in all patients with BTM. This effect was maintained during the five years of treatment (p < 0.001). These findings support the concept that DFX may be even more effective than DFO in improving liver function because of better compliance to treatment. 28,30

Reduction of serum ferritin concentration was associated with a significant decrease in serum ALT, AST and ALP concentrations and increase in IGF-I levels. The significant correlations between serum ferritin concentrations and ALT and AST levels (p < 0.01) suggest that the reduction of hepatic iron load and LIC reduce the hepatic cellular derangement and cell damage and improve liver functions. In support to this view, histological changes of liver biopsy specimens have been shown to correlate significantly with ALT and serum ferritin level.^33^–^34^

Measurement of fibrosis not only helps to stage the severity of disease, it allows serial determination of disease progression. Unfortunately, neither measuring liver enzymes nor IGF-1 can determinate the extent of hepatic fibrosis progression in BMT patients and different outcomes of liver disease may be expected in these patients. A non-invasive method for fibrosis determination (e.g. transient elastography) could add some

Information about liver damage at the end of follow-up, allowing a useful comparison with the evolution of laboratory data and the efficacy of treatment.^35^

In a study on 40 thalassemic children, using the Knodell histological activity index (HAI), 28 children (70%) had 3–4 grade hemosiderosis, 24 (60%) had HAI score between 13/22 to 18/22 and 18 patients (45%) developed cirrhotic changes.^36^ The study done by Li et al. revealed that 30% cases showed HAI stage three and 44% patients showed grade 3–4 hemosiderosis in transfusion dependent BMT children.^8^ Another study by Jean et al. including histological evaluation of liver biopsy in 86 children with thalassemia indicated that some patients developed cirrhosis as early as 7–8 years of age.^3^

IGF-I concentrations were significantly decreased in our BMT patients compared to age and sex published standards. IGF-I concentrations were correlated significantly with serum ALT (r = − 0.26; p = 0.05) suggesting that impaired liver function (increased ALT) may be an important cause of decreased synthesis of hepatic IGF-I in these patients. Negative associations between aspartate aminotransferase (AST) and γ-GT and IGF-1 levels, as well as between AST activity and IGF-1 levels have been detected in patients with chronic liver diseases.^37^

Physiologically, GH stimulates liver IGF-I synthesis and secretion. Therefore, individuals with GH sufficiency should have normal serum IGF-1 level. In thalassemia major, the prevalence of low serum IGF-1 was much higher than that of the GH deficiency.^38^,^39^ The IGF-I response and the linear growth after exogenous administration of GH were less than that seen in GH deficient children treated with GH.^39^,^40^ These data suggest that thalassemic patients had some degree of GH insensitivity. Many factors, other than GH, could control hepatic IGF-I synthesis and circulating IGF-1 level. Patients with chronic liver disease could have low serum IGF-1 despite sufficient GH secretion.^39^–^41^

Hepatic stellate cells are stimulated by insulin-like growth factor 1 (IGF-I) and high IGF-1 levels attenuate fibrogenesis and accelerate liver regeneration. This effect is mainly mediated by up-regulation of hepatic growth factor and down-regulation of transforming growth factor β 1.^42^ Therefore, decreased IGF-1 levels in BMT patients may impair the regeneration and increase the risk for deterioration of function and chronicity. In our study, a significant increase in IGF-I levels as a consequence of decreased ferritin levels after the use of DFX therapy points out to a possible better prognosis of hepatic regeneration and/or improvement of synthetic hepatic functions in these patients.

## Conclusions

In hepatitis-seronegative BMT patients, DFO treatment given at a younger age was associated with mild significant hepatic dysfunction. However, DFX treatment significantly decreased serum ALT, AST and ALP and increased IGF-I concentrations. The positive correlation between serum ferritin and ALT concentrations and the negative correlation between IGF-I concentrations and ferritin and ALT suggest that hepatic iron overload impairs in these patients the hepatic functions and decreases IGF-I synthesis, even in the absence of hepatitis.

## Figures and Tables

**Table 1:** Long-term changes in liver functions in thalassemic patients.

	5 y	6 y	7 y	8 y	9 y	10 y	11 y	12 y	13 y	14 y	15 y	16 y	17 y	18 y
**ALT (U/L)**	53	69	70	63	54	43	41	45	55	40	45	44	48	45
**AST (U/L)**	51	63	57	51	52	71	39	42	45	41	44	45	47	42
**ALP (U/L)**	187	199	1818	225	215	234	214	239	212	231	224	179	130	115
**Albumin (g/dl)**	45	45	44	45	45	44	46	45	46	45	45	46	45	47
**IGF-I (ng/dl)**	33	43	62	68	83	99	77	101	93	102	134	129	175	179
**Bilirubin (μmol/L)**	27	25	28	30	31	33	35	35	38	39	39	42	46	51
**Serum ferritin (μg/L)**	2448	2493	2372	2050	2188	2052	2082	2282	2515	2256	1555	1517	1462	1355

**Table 2:** Correlation between liver functions and ferritin concentrations in thalassemic patients.

	Age	Seum ferritin	IGF-I	ALT	AST	ALP	Albumin	Bilirubin
**Age**	1.00							
**Serum ferritin**	0.17	1.00						
**IGF-I**	0.18	−0.29*	1.00					
**ALT**	−0.35*	0.45*	−0.21	1.00				
**AST**	−0.30*	0.33*	−0.18	0.81*	1.00			
**ALP**	−0.19	−0.16	−0.18	−0.13	0.02	1.00		
**Albumin**	0.08	0.13	0.09	−0.07	−0.07	0.01	1.00	
**Bilirubin**	0.26*	0.15	−0.16	0.00	−0.12	−0.13	0.08	1.00

* p <0.05
